# Investigation of the accuracy of a noninvasive continuous blood pressure device in different age groups and its ability in detecting hypertension and hypotension: an observational study

**DOI:** 10.1186/s12871-019-0899-z

**Published:** 2019-12-05

**Authors:** Ziwei Wang, Guizhen Chen, Kaizhi Lu, Yuan Zhu, Yan Chen

**Affiliations:** Department of Anesthesia, Southwest Hospital, The Third Military Medical University, Chongqing, 400038 China

**Keywords:** Continuous and noninvasive blood pressure, Invasive arterial pressure, Measurement accuracy, Hypertension and hypotension

## Abstract

**Background:**

CNAP monitor is a continuous and noninvasive blood pressure (BP) measurement device that can be used in intraoperative monitoring. But whether its accuracy changes with age and its detectability of hypertension and hypotension are still unclear. This study was to investigate the agreement of CNAP and invasive arterial pressure (IAP) in different age groups, and the ability of CNAP to detect hypertension and hypotension.

**Methods:**

This observational study enrolled 48 Chinese patients undergoing surgery under general anaesthesia, including 25 relatively old patients (age between 50 and 70) and 23 relatively young patients (age between 18 and 49). IAP was monitored at the radial artery and CNAP was applied on the opposite arm simultaneously. Paired BP data in the entire surgery were recorded, and analyzed with Bland Altman plot and Spearman correlation. The ratio of the hypertension and hypotension episodes detected by IAP and CNAP was analyzed using chi-square test.

**Results:**

7990 valid paired BP data were analyzed, wherein 4186 data were from 25 relatively old patients, and the other data were from 23 relatively young patients. Bias (SD) for relatively old patients was: systolic BP (SBP): − 6.5 (18.6) mm Hg; diastolic BP (DBP): 9.3 (7.8) mmHg; and mean BP (MBP): 4.2 (9.5) mm Hg. Bias (SD) for relatively young patients was: SBP: − 6.2 (12.1) mm Hg; DBP: 10.6 (6.9) mmHg; and MBP: 4.8 (7.3) mm Hg. The correlation between CNAP and IAP was higher in MBP than those in SBP and DBP, and it decreased with the increase of age. Comparing to IAP, CNAP tended to miss reporting a high SBP, low DBP and low MBP, and misinform a low SBP, high DBP and high MBP.

**Conclusion:**

CNAP showed acceptable agreement with IAP in MBP for all age groups, but reduced agreement with IAP in SBP and DBP, especially for relatively old patients. Ability of CNAP to detect hypertension and hypotension episodes was weaker than IAP. Therefore, CNAP monitor is suitable for young patients and hemodynamically stable surgery, but may not be recommended for old patients with arteriosclerosis and diabetes or surgeries expecting to have fluctuating blood pressure.

## Background

Perioperative blood pressure is usually measured in two ways: invasive or noninvasive. The invasive arterial line provides continuous monitoring and high accuracy, but it is technically demanding and has risk of complications such as trauma, bleeding, infection, thrombosis, embolism, distal ischemia, and the formation of a pseudoaneurysm [1, 2]. Therefore, it is mostly used in high-risk operations or critically ill patients. The oscillometric pressure device is used in most surgical procedures for blood pressure monitoring due to it is non-invasive and easy to use. But it can only provide intermittent measurement (e.g., every 3-5 min), may not reflect the changes in blood pressure timely and effectively. Inadequate diagnosis and delayed treatment of the changes in blood pressure may increase postoperative complications and mortality [3–5].

Recently, a new monitor CNAP™ Monitor 500 (CNSystems Medizintechnik AG, Graz, Austria) for continuous and noninvasive blood pressure measurement has been used in intraoperative and emergency monitoring [6–10]. The CNAP system is based on the principle of volume clamped method developed by Peňáz in 1973 [11]. It can detect continuous pressure wave by a double-finger cuff with photoplethysmography and a pressure transducer fixed on the forearm. An NIBP cuff is launched intermittently for calibration.

Previous studies reported the comparison of CNAP monitor with arterial line and oscillometric pressure device in general anesthesia or spinal anesthesia [6–9]. The results indicated that CNAP monitor showed an acceptable agreement with invasive and oscillometric pressure for mean arterial pressure (MAP). In addition, CNAP monitor could detect more hypotensive episodes than oscillometric device. However, two points have not been reported: first, whether the accuracy of the technique is different for different age groups; Second, promptness of the device to detect extreme blood pressure (extreme high or low blood pressure) in comparing to arterial line.

The aim of this study was to investigate the accuracy difference of CNAP monitor in the relatively old and relatively young people, as well as the ability of the CNAP monitor detecting the hypertension and hypotension episodes, by taking arterial line as a reference.

## Methods

### Participants

The observational monocentric study was conducted at department of anesthesia, Southwest Hospital in Chongqing, China, and followed the STROBE statements [12]. This study was approved by Ethics Committee of the First Affiliated Hospital of Third Military Medical University, PLA (Scientific Research No. 382014). After obtaining the written informed consents, 50 Chinese patients undergoing elective abdominal, open chest thoracic and neurological surgeries were recruited into this study. Inclusion criteria were a clinical requirement of invasive arterial pressure measurement, American Society of Anesthesiologists (ASA) Classification between 1 and 3, age between 18 and 70, and the expected time of surgery > 3 h. Exclusion criteria were emergency surgery, BMI < 17 kg/m^2^ or > 30 kg/m^2^, a positive Allen’s test, severe cardiopulmonary disease, peripheral vascular disease or preexisting edema of the upper limb. 2 patients were dropped out due to failing to place the arterial line into the radial artery. 48 patients were eventually enrolled into this study, in which, 25 patients belonged to relatively old group (age between 50 and 70) and 23 patients belonged to relatively young group (age between 18 and 49).

### Blood pressure measurement and collection

After the patients were admitted to the operation room, ECG and SpO2 were measured routinely. Before induction of anesthesia, a 20 G, 32-mm-long catheter (B Braun Melsungen AG, Melsungen Germany) was placed into radial artery, and connected to a disposable pressure transducer (B Braun Melsungen AG, Melsungen Germany). The transducer was positioned at the patient’s midaxillary line and zeroed to atmospheric pressure. The damping coefficient and natural frequency were evaluated using the fast flush method as described by Gardner [13]. After invasive arterial pressure (IAP) measurement was established, the CNAP™ Monitor with a NIBP cuff on the upper arm and two finger cuffs on the index and middle fingers was applied to the opposite side to the arterial line. Suitable size of the finger cuff was chosen according to the finger diameter. Calibration time was set to 30 mins.

Following pre-oxygenation, general anesthesia was induced with intravenous propofol 2 mg/kg, fentanyl 5 μg/kg and vecuronium 0.1 mg/kg. After tracheal intubation, the lungs were ventilated (tidal volume 8 ml/kg, plateau pressure 5-13cmH2O) to maintain SpO2 above 96% and PETCO2 between 32 and 38 mmHg. Anaesthesia was maintained with inhalation of sevoflurane and the minimum alveolar concentration (MAC) was kept between 0.8 and 1.2. Remifentanyl was infused with target effect-site concentration 3 ng/ml. Blood pressure was supported by pharmacological vasodilatation (nitroglycerine 0.5–1.0μg/kg/min) or vasoconstriction (ephedrine 5-10 mg/dose and norepinephrine 0.03–0.5μg/kg/min). The titration speed of the crystal solution during anesthesia was 8-15 ml/kg/h. Blood transfusion was performed when the hemoglobin concentration was less than 80 g/L.

Blood pressure including systolic, diastolic and mean (SBP, DBP, and MBP) from the CNAP™ device and the arterial line were automatically recorded into a computer using an anesthesia information acquisition system (DoCare; Medical Treatment Technology Co. Ltd., Beijing, China) every two minutes throughout the surgery (from anesthesia induction to skin suture). Before data analysis, blood pressure artifacts, such as arterial blood sampling and patient repositioning, were detected and removed from the data using Matlab software (Matlab 7.0; Math Works Inc., Natick, Massachusetts, USA).

### Statistical analysis

The sample size was chosen based on the recommendations in the protocol of the Association for the Advancement of Medical Instrumentation (AAMI) that at least 15 patients should be included when comparing to arterial line [14]. We included 25 and 23 patients for relatively old group and relatively young group respectively, resulting in a statistical power of 90%.

Statistical analyses were carried out using Graph Pad Prism v5.0 (Graph Pad Software Inc., San Diego, California, USA) and IBM SPSS Statistics 21 (SPSS Inc., Chicago, IL, USA). Bland–Altman plots for repeated measures [15–17] were used to analyze SBP, DBP, and MBP data collected from CNAP™ device and the arterial line. Bias was the mean difference between IAP and CNAP. Limits of agreement were the range including 95% of the differences between IAP and CNAP. Spearman correlation was used to characterize the relation between CNAP and IAP. The ratio of the hypertension and hypotension episodes detected by IAP and CNAP was analyzed using chi-square test. Data were expressed as mean (SD) unless stated otherwise. A *P*-value of 0.05 was considered statistically significant.

## Results

Patient characteristics are summarized in Table 1. A total of 7990 valid, paired CNAP and IAP readings, with 150–180 readings per patient, were used for analysis, wherein, there were 4186 readings from relatively old group, and the others were from relatively young group.

**Table 1:** Patient characteristic data (total, *n* = 48; relatively old, *n* = 25; relatively young, *n* = 23)

	Total	Relatively Old	Relatively Young
Age	48 (19–68)	60 (54–68)	37 (19–48)
Gender (M/F)	26/22	14/11	12/11
BMI (kg. m^−2^)	22.4 (3.8)	22.8 (4.7)	21.9 (2.9)
ASA (I/II/III)	3/28/17	0/15/10	3/13/7
Surgery (abd/ thor/ neuro)	27/11/10	15/5/5	12/6/5
Hypertension History	6	4	2
Diabetic History	3	3	0

Data are mean (SD), mean (range) for age, or absolute numbers. Abd, abdominal surgery; thor, thoracic surgery; neuro, neurological surgery

**Fig. 1:**
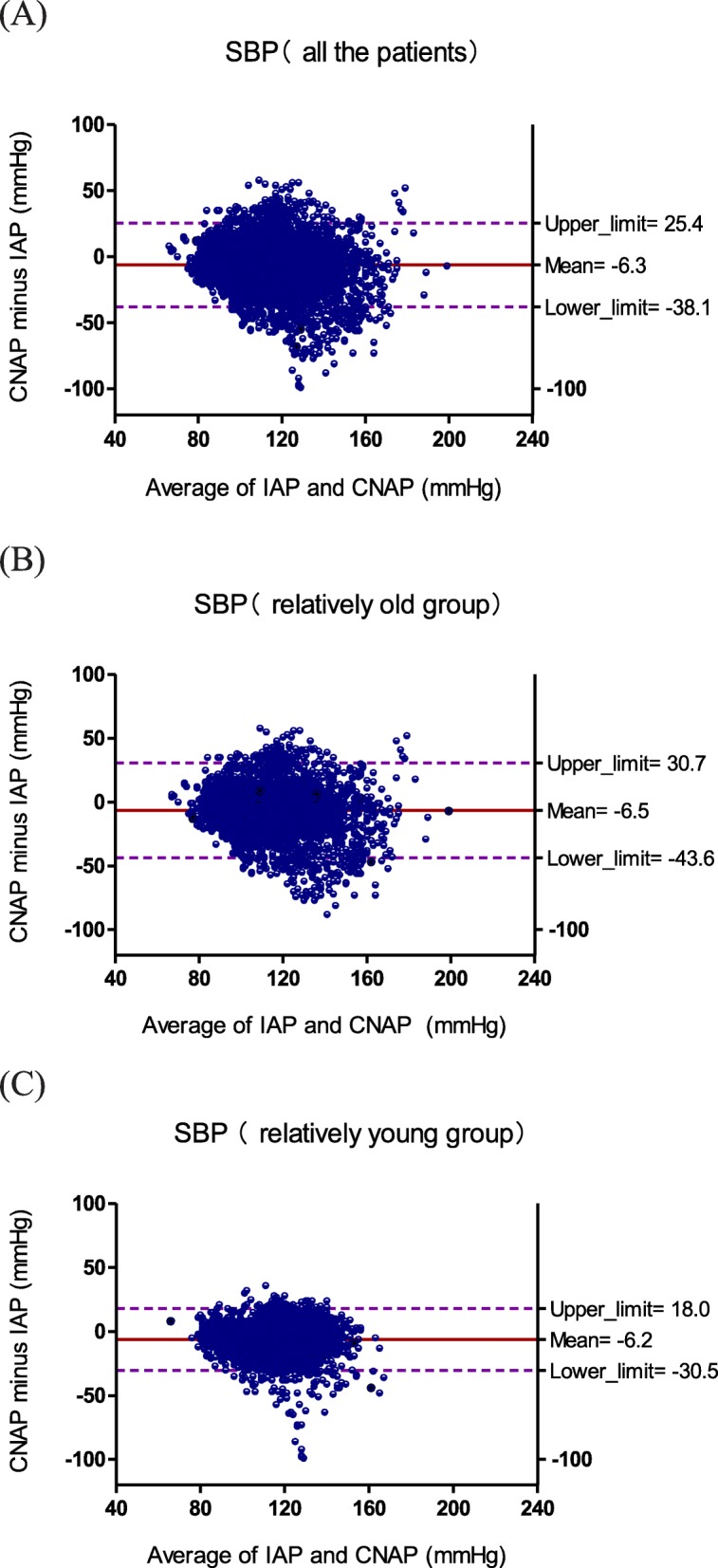
Bland–Altman plots for repeated measurements of SBP for (**a**) all the patients, (**b**) relatively old group, (**c**) relatively young group

**Fig. 2:**
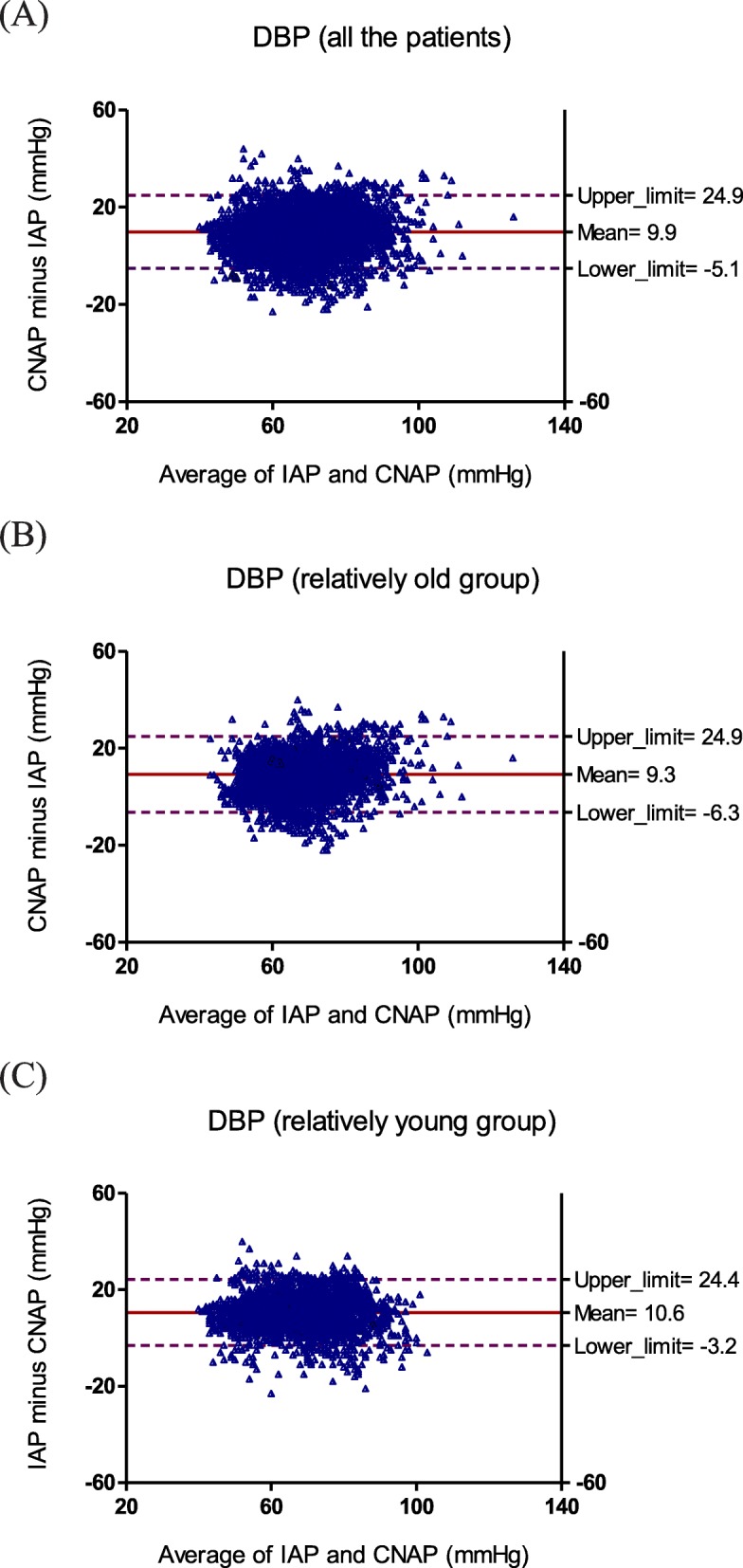
Bland–Altman plots for repeated measurements of DBP for (**a**) all the patients, (**b**) relatively old group, (**c**) relatively young group

**Fig. 3:**
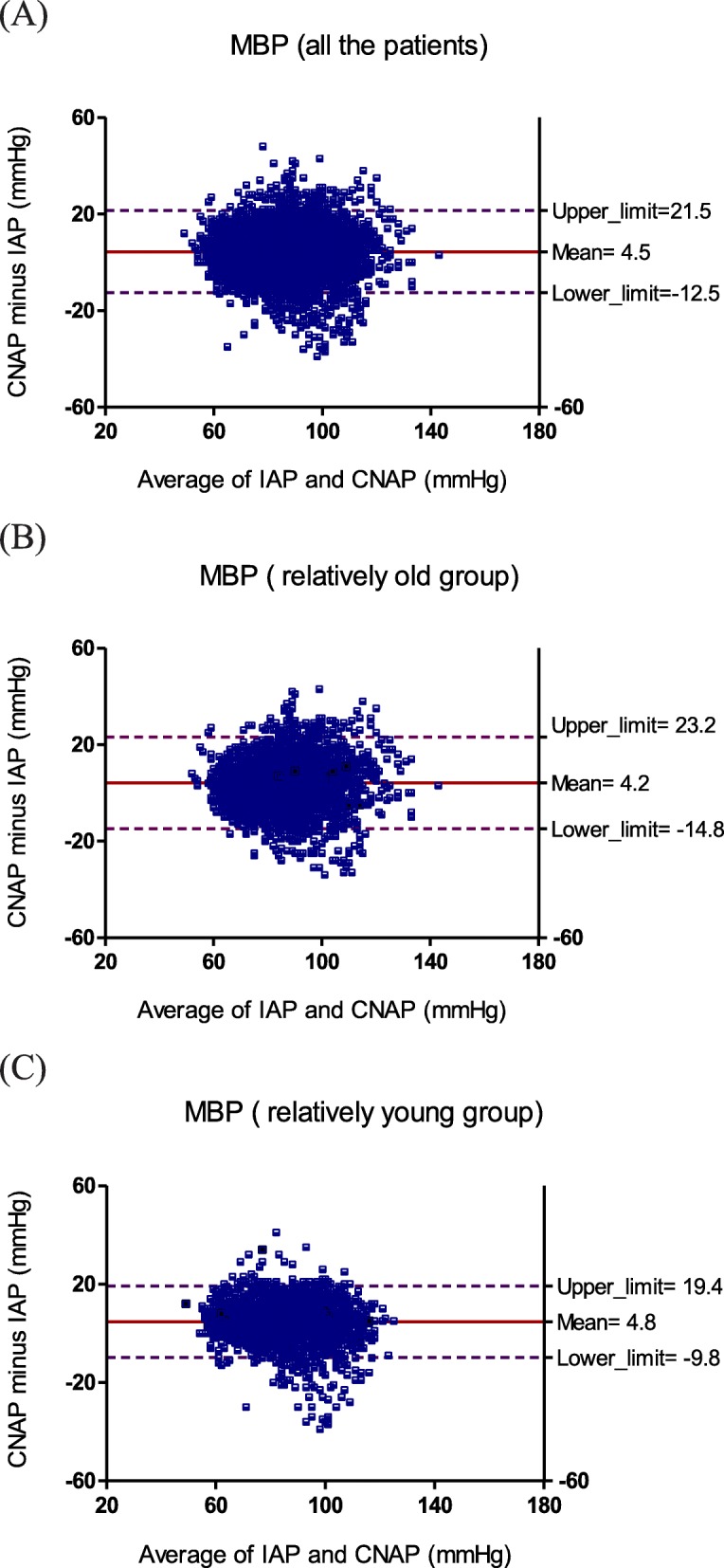
Bland–Altman plots for repeated measurements of MBP for (**a**) all the patients, (**b**) relatively old group, (**c**) relatively young group

The Bland–Altman plots for SBP, DBP, and MBP were calculated for all the patients (Fig. 1), the relatively old group (Fig. 2) and the relatively young group (Fig. 3). For SBP of all the patients, the bias (SD of bias) was − 6.3 (15.9) mmHg (limits of agreement: − 38.1 to 25.4 mmHg) reflecting that the CNAP underestimated IAP; For SBP of relatively old group, the bias (SD of bias) was − 6.5 (18.6) mmHg (limits of agreement: − 43.6 to 30.7 mmHg) reflecting that CNAP underestimated IAP and the degree of deviation is higher; For SBP of relatively young group, the bias (SD of bias) was − 6.2 (12.1) mmHg (limits of agreement: − 30.5 to 18.0 mmHg) reflecting that CNAP underestimated IAP and the degree of deviation is lower.

For DBP, the bias (SD of bias) was 9.9 (7.5) mmHg (limits of agreement: − 5.1 to 24.9 mmHg) for all the patients, 9.3 (7.8) mmHg (limits of agreement: − 6.3 to 24.9 mmHg) for the relatively old group, and 10.6 (6.9) mmHg (limits of agreement: − 3.2 to 24.4 mmHg) for the relatively young group, reflecting that CNAP overestimated IAP, and the degree of deviation was consistent in all age.

For MBP, the bias (SD of bias) was 4.5 (8.5) mmHg (limits of agreement: − 12.5 to 21.5 mmHg) for all the patients, 4.2 (9.5) mmHg (limits of agreement: − 14.8 to 23.2 mmHg) for the relatively old group, and 4.8 (7.3) mmHg (limits of agreement: − 9.8 to 19.4 mmHg) for the relatively young group, reflecting that CNAP slightly overestimated IAP, and the degree of deviation was slightly higher for the relatively old group and slightly lower for the relatively young group.

Spearman Correlation between CNAP and IAP for different age groups are summarized in Table 2. The correlation coefficients of MBP were higher than those of SBP and DBP for all age of patients. The correlation coefficients of SBP、DBP and MBP decreased with the increase of age, and the 95% confidence intervals became wider with the increase of age.

**Table 2:** Spearman correlation between CNAP and IAP

Age group	Spearman r (95% confidence interval)		
	SBP	DBP	MBP
Total	0.66 (0.65–0.67)	0.77 (0.76–0.78)	0.81(0.80–0.82)
Relatively Old	0.60 (0.58–0.62)	0.71 (0.69–0.73)	0.75 (0.73–0.76)
Relatively Young	0.75 (0.74–0.77)	0.83 (0.82–0.84)	0.87 (0.87–0.88)

**Fig. 4:**
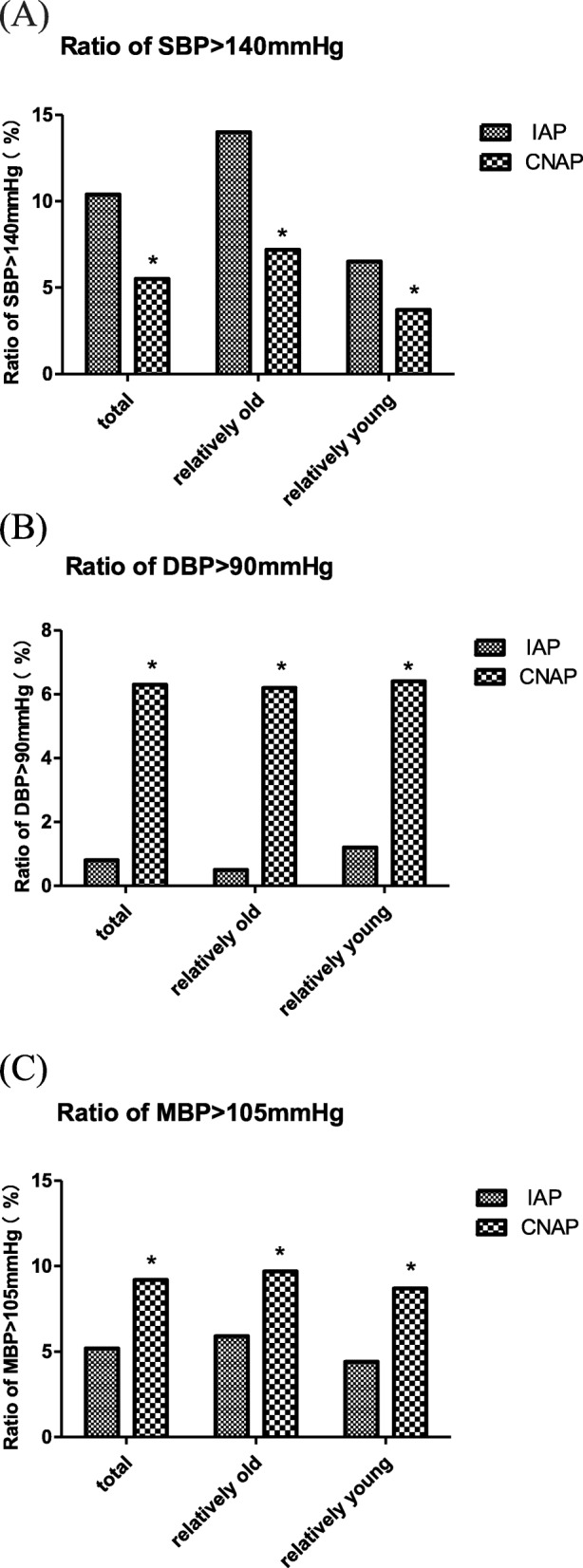
Ratio of hypertension episode ((**a**) SBP > 140 mmHg, (**b**) DBP > 90 mmHg and (**c**) MBP > 105 mmHg) detected by IAP and CNAP. Ratio is the quotient of the number of hypertension readings divided by the total number of readings, expressed as a percentage. * indicates *p* < 0.01 when IAP is compared to CNAP using chi-square test

**Fig. 5:**
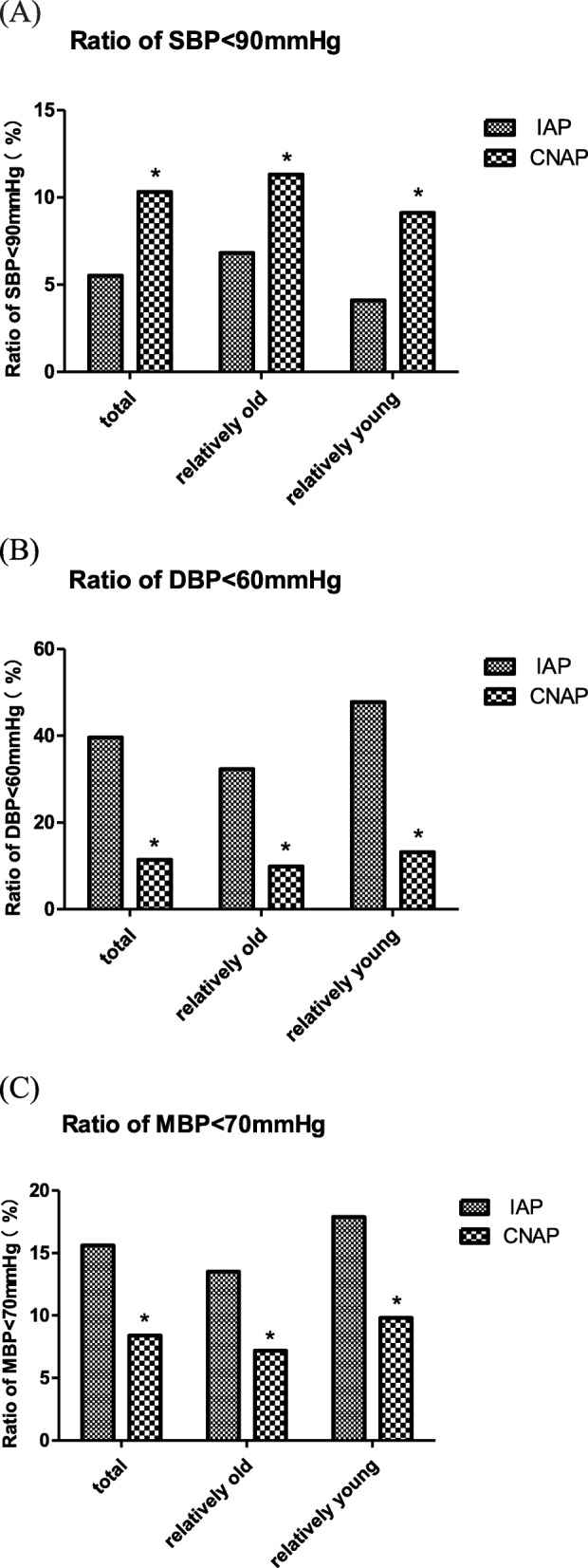
Ratio of hypotension episode ((**a**) SBP < 90 mmHg, (**b**) DBP < 60 mmHg, (**c**) MBP < 70 mmHg) detected by IAP and CNAP. Ratio is the quotient of the number of hypotension readings divided by the total number of readings, expressed as a percentage * indicates p < 0.01 when IAP is compared to CNAP using chi-square test.

Ratio of hypertension episode (SBP > 140 mmHg, DBP > 90 mmHg and MBP > 105 mmHg) and hypotension episode (SBP < 90 mmHg, DBP < 60 mmHg and MBP < 70 mmHg) detected by IAP and CNAP were shown in Fig. 4 and Fig. 5, respectively.

For SBP, the hypertension episode (SBP > 140 mmHg) detected by CNAP was significant less than that detected by IAP in all the patients (5.5% vs 10.4%), the relatively old group (7.2% vs 14.0%) and the relatively young group (3.7% vs 6.5%); but the hypotension episodes (SBP < 90 mmHg) detected by CNAP was significant more than that detected by IAP in all the patients (10.3% vs 5.5%), the relatively old group (11.3% vs 6.8%) and the relatively young group (9.1% vs 4.1%), which indicated that CNAP tended to miss reporting high SBP and misinform low SBP.

For DBP, the hypertension episode (DBP > 90 mmHg) detected by CNAP was significant more than that detected by IAP in all the patients (6.3% vs 0.8%), the relatively old group (6.2% vs 0.5%) and the relatively young group (6.4% vs 1.2%); but the hypotension episodes (DBP < 60 mmHg) detected by CNAP was significant less than that detected by IAP in all the patients (11.4% vs 39.6%), the relatively old group (9.8% vs 32.3%) and the relatively young group (13.1% vs 47.8%), which indicated that CNAP tended to misinform high DBP and miss reporting low DBP.

For MBP, the hypertension episode (MBP > 105 mmHg) detected by CNAP was significant more than that detected by IAP in all the patients (9.2% vs 5.2%), the relatively old group (9.7% vs 5.9%) and the relatively young group (8.7% vs 4.4%); but the hypotension episodes (MBP < 70 mmHg) detected by CNAP was significant less than that detected by IAP in all the patients (8.4% vs 15.6%), the relatively old group (7.2% vs 13.5%) and the relatively young group (9.8% vs 17.9%), which indicated that CNAP tended to misinform high MBP and miss reporting low MBP.

## Discussion

We investigated the agreement of CNAP monitor with IAP in the relatively old and relatively young groups respectively, as well as the reaction capacity of CNAP monitor to hypertension and hypotension. The main findings of our study were: (i) CNAP showed an acceptable agreement with IAP for MBP, but a big positive bias for DBP, a smaller pulse pressure difference, and a high degree of deviation (SD of bias) for SBP; (ii) the biases for SBP, DBP and MBP displayed no age-related changes, but the degree of deviation (SD of bias) for SBP is higher in the relatively old groups than that in the relatively young groups; (iii) The correlation between CNAP and IAP was relatively higher for MBP but relatively lower for SBP and DBP, and it decreased with the increase of age. (iv) Ability of CNAP monitor to detect hypertension and hypotension was weaker than that of IAP, CNAP tended to miss reporting a high SBP, low DBP and low MBP, and misinform a low SBP, high DBP and high MBP.

MBP, SBP and DBP have their respective roles and significance in clinical practice. MBP is an important index for clinical anesthesia in evaluating the perfusion of vital organs such as kidneys and heart [18]. SBP has important clinical significance in the diagnosis of hypertension, especially isolated systolic hypertension (increased SBP with normal DBP and MBP). Increased SBP alone is associated with increased risk of stroke and coronary events [19]. DBP is an indicator reflecting the change of peripheral resistance during surgery, because it shows a better correlation with peripheral resistance than SBP and MBP. It also determines coronary perfusion pressure. Maintaining an appropriate DBP is important for coronary perfusion, especially in patients with coronary heart disease. Regarding to MBP, CNAP provided a bias (SD of bias) of 4.5 (8.5) mmHg for all the patients in compared with IAP, which was very closed to the accuracy criteria of AAMI (bias≤5 mmHg and SD of bias≤8 mmHg) [14]. So the reliability of MBP measured by CNAP could be considered as clinically acceptable. However, comparing to IAP, CNAP showed a high degree of deviation for SBP measurement especially in elder patients and tended to miss detecting a high SBP, which might lead to misdiagnosis, missed diagnosis and improper medication of intraoperative hypertension. For DBP, CNAP invariably overestimated IAP and tended to miss detecting a low DBP, which might result in delayed or missed use of vasoactive drugs and increased complications due to insufficient intraoperative coronary perfusion.

The reason for a larger SBP deviation in relatively old patients than that in relatively young group may be the arterial compliance and structural changes caused by physiological or pathological arteriosclerosis and diabetes. Atherosclerosis increases measurement variability can be explained in two aspects. In the first aspect, the loss of compliance of the artery walls due to atherosclerosis increases the pulse wave amplification, which results in a higher SBP and a larger pulse pressure difference in distal artery than that in proximal artery [20, 21]. In addition, the pathological arteriosclerosis due to diabetes etc. may result in arterial thrombosis, narrowed or blocked arteries, and consequently, distorted arterial pulse wave and unpredictable changes in SBP [22, 23]. CNAP monitor is calibrated according to brachial arterial pressure, and for most relatively old patients who usually suffer from physiological or pathological arteriosclerosis and diabetes. There may be a big inherent difference between radial and brachial blood pressures, especially in SBP. But for relatively young patients who are usually not affected with arteriosclerosis and diabetes, the difference in SBP between different parts of the body is relatively small. In the second aspect, arteriosclerosis reduces the accuracy of oscillometry, which is the technique used for calibration of CNAP monitor. Other studies had indicated that the oscillometric measurement showed disagreement with sphygmomanometer on the presence of arterial stiffness and diabetes [24, 25].

Compared with IAP, CNAP provided a lower SBP and a higher DBP, and thus a smaller pulse pressure difference (SBP minus DBP), which may be caused by the measurement principle of CNAP monitor. In the process of measurement, continuous pressure is applied on a finger by a ring cuff, as the time prolongs, venous blood backflow in finger is hindered, resulting in blood accumulating in the finger, which leads to a decrease in the amplitude of arterial pulse wave and the pulse pressure difference. Although CNAP monitor exchanged measurement fingers every half an hour and periodically calibrated the measurement value by oscillometry, the blood accumulation in finger caused by continuous pressure might still affect the accuracy of blood pressure measurement and the ability to respond to changes in blood pressure. This may be one of the reasons for the poor performance of CNAP in hypertension and hypotension conditions.

Our study has some limitations: (i) We did not evaluate the results according to the recommendations of the protocol of the AAMI, because this standard is applied when comparing blood pressure measured from noninvasive intermittent devices such as oscillometry with invasive arterial pressure. So far, there has been no guideline or agreement regarding accuracy criteria of continuous and noninvasive devices compared with invasive blood pressure. (ii) The present study did not focus on the influence of anesthetics, analgesics and vasoactive drugs on the performance of CNAP monitor. However, the readings during pharmacological interventions, although not specifically identified, were included in the analysis. A different study design should clarify this issue. (iii) In this study, the calibration interval is 30 min according to the default set of CNAP monitor, but it seems too long to ensure the accuracy in CNAP. The optimal calibration interval in the course of general anesthesia has not been investigated, which requires further studies. (iv) The CNAP monitor was used on the opposite arm to the arterial line, the blood pressure difference between the left and right arms may contribute to bias. (v) Manual calibration was not performed in the case of repositioning of patients and special surgical procedures, which may contribute to bias. (vi) We did not evaluate the measurement results according to different types of surgery. A different study design with bigger sample size may clarify this issue.

## Conclusion

In conclusion, CNAP provides a beat-to-beat readings, a visual representation of the pulse wave, and an acceptable agreement with invasive arterial measurements in mean blood pressure for all age groups. Nevertheless, its precisions in systolic and diastolic pressures are reduced, especially in older patients with arteriosclerosis and diabetes. Also it is less capable of detecting hypertension and hypotension episodes in comparison to intra-arterial pressure measurement. Therefore, CNAP monitor may be suitable for relatively young patients with less arteriosclerosis and diabetes and hemodynamically stable surgery, but should be prudent to use in old patients with high risk or surgical patients who are expected to have fluctuating blood pressure.

## Data Availability

Because of the secrecy policy restrictions on military university, the datasets used in this study are not allowed to be deposited in publicly. The datasets are available from the corresponding author on reasonable request.

## Abbreviations

AAMI: Association for the Advancement of Medical Instrumentation
ASA: American Society of Anesthesiologists
BP: Blood pressure
DBP: Diastolic blood pressure
IAP: Invasive arterial pressure
MBP: Mean blood pressure
SBP: Systolic blood pressure
